# A machine learning approach to define antimalarial drug action from heterogeneous cell-based screens

**DOI:** 10.1126/sciadv.aba9338

**Published:** 2020-09-25

**Authors:** George W. Ashdown, Michelle Dimon, Minjie Fan, Fernando Sánchez-Román Terán, Kathrin Witmer, David C. A. Gaboriau, Zan Armstrong, D. Michael Ando, Jake Baum

**Affiliations:** 1 Department of Life Sciences, Imperial College London, London, UK.; 2 Google Research, Applied Science Team.; 3 Facility for Imaging by Light Microscopy, Imperial College London, London, UK.

## Abstract

Drug resistance threatens the effective prevention and treatment of an ever-increasing range of human infections. This highlights an urgent need for new and improved drugs with novel mechanisms of action to avoid cross-resistance. Current cell-based drug screens are, however, restricted to binary live/dead readouts with no provision for mechanism of action prediction. Machine learning methods are increasingly being used to improve information extraction from imaging data. These methods, however, work poorly with heterogeneous cellular phenotypes and generally require time-consuming human-led training. We have developed a semi-supervised machine learning approach, combining human- and machine-labeled training data from mixed human malaria parasite cultures. Designed for high-throughput and high-resolution screening, our semi-supervised approach is robust to natural parasite morphological heterogeneity and correctly orders parasite developmental stages. Our approach also reproducibly detects and clusters drug-induced morphological outliers by mechanism of action, demonstrating the potential power of machine learning for accelerating cell-based drug discovery.

## Teaser

Machine learning is applied to high-throughput microscopy images of malaria parasites to define antimalarial drug mode of action.

## INTRODUCTION

Cell-based screens have substantially advanced our ability to find new drugs (*1*). However, most screens are unable to predict the mechanism of action (MoA) of identified hits, necessitating years of follow-up after discovery. In addition, even the most complex screens frequently find hits against cellular processes that are already targeted (*2*). Limitations in finding new targets are becoming especially important in the face of rising antimicrobial resistance across bacterial and parasitic infections. This rise in resistance is driving increasing demand for screens that can intuitively find new antimicrobials with novel MoAs. Demand for innovation in drug discovery is exemplified in efforts on targeting *Plasmodium falciparum*, the parasite that causes malaria. Malaria continues to be a leading cause of childhood mortality, killing nearly half a million children each year (*3*). Drug resistance has emerged to every major antimalarial to date including rapidly emerging resistance to frontline artemisinin-based combination therapies (*4*). While there is a healthy pipeline of developmental antimalarials, many target common processes (*5*) and may therefore fail quickly because of prevalent cross-resistance. Thus, solutions are urgently sought for the rapid identification of new drugs that have a novel MoA at the time of discovery.

Parasite cell morphology within the human contains inherent MoA-predictive capacity. Intracellular parasite morphology can distinguish broad stages along the developmental continuum of the asexual parasite (responsible for all disease pathology). This developmental continuum includes early development (early and late ring form), feeding (trophozoite), genome replication or cell division (schizont), and extracellular emergence [merozoite; see (*6*) for definitions]. Hence, drugs targeting a particular stage should manifest a break in the continuum. Morphological variation in the parasite cell away from the continuum of typical development may also aid drug MoA prediction if higher information granularity can be generated during a cell-based screen. Innovations in automated fluorescence microscopy have markedly expanded available data content in cell imaging (*7*). By using multiple intracellular markers, an information-rich landscape can be generated from which morphology, and, potentially, drug MoA can be deduced. This increased data content is, however, currently inaccessible both computationally and because it requires manual expert-level analysis of cell morphology. Thus, efforts to use cell-based screens to find drugs and define their MoA in a time-efficient manner are still limited.

Machine learning (ML) methods offer a powerful alternative to manual image analysis, particularly deep neural networks (DNNs) that can learn to represent data succinctly. To date, supervised ML has been the most successful application for classifying imaging data, commonly based on binning inputs into discrete, human-defined outputs. Supervised methods using this approach have been applied to study mammalian cell morphologies (*8*, *9*) and host-pathogen interactions (*10*). However, discrete outputs are poorly suited for capturing a continuum of morphological phenotypes, such as those that characterize either malaria parasite development or compound-induced outliers, since it is difficult or impossible to generate labels of all relevant morphologies a priori. A cell imaging approach is therefore needed that can function with minimal discrete human-derived training data before computationally defining a continuous analytical space, which mirrors the heterogeneous nature of biological space.

Here, we have created a semi-supervised model that discriminates diverse morphologies across the asexual life cycle continuum of the malaria parasite *P. falciparum.* By receiving input from a deep metric network (*11*) trained to represent similar consumer images as nearby points in a continuous coordinate space (an embedding), our DNN can successfully define unperturbed parasite development with a much finer information granularity than human labeling alone. The same DNN can quantify antimalarial drug effects both in terms of life cycle distribution changes [e.g., killing specific parasite stage(s) along the continuum] and morphological phenotypes or outliers not seen during normal asexual development. Combining life cycle and morphology embeddings enabled the DNN to group compounds by their MoA without directly training the model on these morphological outliers. This DNN analysis approach toward cell morphology therefore addresses the combined needs of high-throughput cell-based drug discovery that can rapidly find new hits and predict MoA at the time of identification.

## RESULTS

**Fig. 1:**
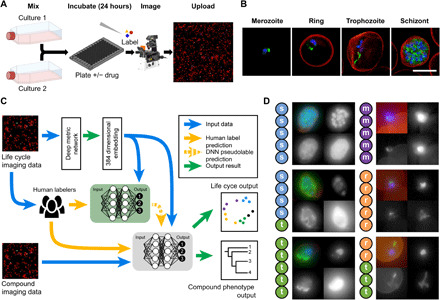
Experimental workflow. (**A**) To ensure all life cycle stages were present during imaging and analysis, two transgenic malaria cultures, continuously expressing sfGFP, were combined (see Materials and Methods); these samples were incubated with or without drugs before being fixed and stained for automated multichannel high-resolution, high-throughput imaging. Resulting datasets (**B**) contained parasite nuclei (blue), cytoplasm (not shown), and mitochondrion (green) information, as well as the RBC plasma membrane (red) and brightfield (not shown). Here, canonical examples of a merozoite, ring, trophozoite, and schizont stage are shown. These images were processed for ML analysis (**C**) with parasites segregated from full field of views using the nuclear stain channel, before transformation into embedding vectors. Two networks were used; the first (green) was trained on canonical examples from human-labeled imaging data, providing ML–derived labels (pseudolabels) to the second semi-supervised network (gray), which predicted life cycle stage and compound phenotype. Example images from human-labeled datasets (**D**) show that disagreement can occur between human labelers when categorizing parasite stages (s, schizont; t, trophozoite; r, ring; m, merozoite). Each thumbnail image shows (from top left, clockwise) merged channels, nucleus staining, cytoplasm, and mitochondria. Scale bar, 5 μm.

Using ML, we set out to develop a high-throughput, cell-based drug screen that can define cell morphology and drug MoA from primary imaging data. From the outset, we sought to embrace asynchronous (mixed stage) asexual cultures of the human malaria parasite, *P. falciparum,* devising a semi-supervised DNN strategy that can analyze fluorescence microscopy images. The workflow is summarized in Fig. 1 (A to C).

### Overview of the approach: Gathering human labels

The *P. falciparum* life cycle commences when free micron-sized parasites (called merozoites; Fig. 1B, far left) target and invade human RBCs. During the first 8 to 12 hours after invasion, the parasite is referred to as a “ring,” describing its diamond ring–like morphology (Fig. 1B, left). The parasite then feeds extensively (trophozoite stage; Fig. 1B, right), undergoing rounds of DNA replication and eventually divides into ~20 daughter cells (the schizont-stage; Fig. 1B, far right), which precedes merozoite release back into circulation (*6*). This discrete categorization belies a continuum of morphologies between the different stages.

The morphological continuum of asexual development represents a challenge when teaching ML models, as definitions of each stage will vary between experts (Fig. 1D and fig. S1). To embrace this, multiple human labels were collected. High-resolution three-dimensional (3D) images of a 3D7 parasite line continuously expressing superfolder green fluorescent protein (sfGFP) in the cytoplasm (3D7/sfGFP) were acquired using a widefield fluorescence microscope (see Materials and Methods), capturing brightfield DNA [4′,6-diamidino-2-phenylindole (DAPI), cytoplasm (constitutively expressed sfGFP), mitochondria (MitoTracker abbreviated subsequently to MITO)], and the RBC membrane [fluorophore-conjugated wheat germ agglutinin (WGA)]. 3D datasets were converted to 2D images using maximum intensity projection. Brightfield was converted to 2D using both maximum and minimum projection, resulting in six channels of data for the ML. Labels (5382) were collected from human experts, populating the categories of ring, trophozoite, schizont, merozoite, cluster-of-merozoites (multiple extracellular merozoites attached after RBC rupture), or debris. For initial validation and as a test of reproducibility between experts, an additional 448 parasites were collected, each labeled by five experts (Fig. 1D).

As demonstrated (Fig. 1D and fig. S1A), human labelers show some disagreement, particularly between ring and trophozoite stages. This disagreement is to be expected, with mature ring stage and early trophozoite stage images challenging to define even for experts. When comparing the human majority vote versus the model classification (fig. S1B and note S1), some disagreement was seen, particularly for human-labeled trophozoites being categorized as ring stages by the ML algorithm.

### Overview of the approach: Learning a data-driven representation of malaria phenotypes

Image patches containing parasites within the RBC or after merozoite release were transformed into input embeddings using the deep metric network architecture originally trained on consumer images (*11*) and previously shown for microscopy images (*12*). Embeddings are vectors of floating point numbers representing a position in high-dimensional space, trained so related objects are located closer together. For our purposes, each image channel was individually transformed into an embedding of 64 dimensions before being concatenated to yield one embedding of 384 dimensions per parasite image.

Embeddings generated from parasite images were next transformed using a two-stage workflow to represent either on-cycle (for mapping the parasite life cycle continuum) or off-cycle effects (for mapping morphology or drug induced outliers). Initially, an ensemble of fully connected two-layer DNN models was trained on the input embeddings to predict the categorical human life cycle labels for dimethyl sulfoxide (DMSO) controls. DMSO controls consisted of the vehicle liquid for drug treatments (DMSO) being added to wells containing no drugs. For consistency, the volume of DMSO was normalized in all wells to 0.5%. This training gave the DNN robustness to control for sample heterogeneity and, hence, sensitivity for identifying unexpected results (outliers). The ensemble was built from three pairs of fully supervised training conditions (six total models). Models only differed in the training data they received. Each network pair was trained on separate (nonoverlapping) parts of the training data, providing an unbiased estimate of the model prediction variance.

After initial training, the supervised DNN predicted its own labels (i.e., pseudolabels) for previously unlabeled examples. As with human-derived labels, DNN pseudolabeling was restricted to DMSO controls (with high confidence) to preserve the model’s sensitivity to off-cycle outliers (which would not properly fit into on-cycle outputs). High confidence was defined as images given the same label prediction from all six models and when all models were confident of their own prediction (defined as twice the probability of selecting the correct label at random). This baseline random probability is a fixed number for a dataset or classification and provided a suitable baseline for model performance.

A new ensemble of models was then trained using the combination of human-derived labels and DNN pseudolabels. The predictions from this new ensemble were averaged to create the semi-supervised model.

### Validation of the *P. falciparum* life cycle continuum

**Fig. 2:**
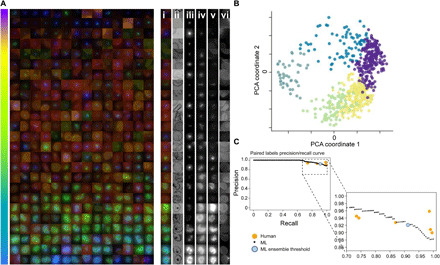
ML continuum of parasite stages. After learning from canonical human-labeled parasite images (for examples, please see Fig. 1B) and filtering debris and other outliers, the remaining life cycle data from asynchronous cultures was successfully ordered by the model. The parasites shown are randomly selected DMSO control parasites from multiple imaging runs, sorted by Angle PCA (**A**). The colored, merged images show RBC membrane (red), mitochondria (green), and nucleus (blue). For a subset of parasites on the right, the colored, merged image plus individual channels are shown: (i) merged, (ii) brightfield minimum projection, (iii) nucleus, (iv) cytoplasm, (v) mitochondria, and (vi) RBC membrane (brightfield maximum projection was also used in ML but is not shown here). The model sorts the parasites in life cycle stage order, despite heterogeneity of signal due to nuances such as imaging differences between batches. The order of the parasites within the continuum seen in (A) is calculated from the angle within the circle created by projecting model outputs using PCA, creating a 2D scatterplot (**B**). This represents a progression through the life cycle stages of the parasite, from individual merozoites (purple) to rings (yellow), trophozoites (green), schizonts (dark green), and finishing with a cluster of merozoites (blue). The precision-recall curve (**C**) shows that human labelers and the model have equivalent accuracy in determining the earlier/later parasite in pairs. The consensus of the human labelers was taken as ground truth, with individual labelers (orange) agreeing with the consensus on 89.5 to 95.8% of their answers. Sweeping through the range of “too close to call” values with the ML model yields the ML curve shown in black. Setting this threshold to 0.11 radians, the median angle variance across the individual models used in the ensemble yields the blue dot.

The semi-supervised model was first used to represent the normal (on-cycle) life cycle continuum. We selected the subset of dimensions in the unnormalized final prediction layer that corresponded to merozoites, rings, trophozoites, and schizonts. This was projected into 2D space using principal components analysis (PCA) and shifted such that its centroid was at the origin. This resulted in a continuous variable where angles represent life cycle stage progression, referred to as Angle-PCA. This Angle-PCA approach permitted the full life cycle to be observed as a continuum with example images despite data heterogeneity (Fig. 2A and fig. S2) and 2D projection (Fig. 2B) following the expected developmental order of parasite stages. This ordered continuum manifested itself without specific constraints being imposed, except those provided by the categorical labels from human experts (see note S2).

To validate the accuracy of the continuous life cycle prediction, pairs of images were shown to human labelers to define their developmental order (earlier/later) with the earliest definition being the merozoite stage. Image pairs assessed also included those considered indistinguishable (i.e., “too close to call”). Of the 295 pairs selected for labeling, 276 measured every possible pairing between 24 parasites, while the remaining 19 pairs were specifically selected to cross the trophozoite/schizont boundary. Human expert agreement with the majority consensus was between 89.5 and 95.8% (note S3), with parasite pairs called equal (too close to call) to 25.7 to 44.4% of the time. These paired human labels had more consensus than the categorical (merozoite, ring, trophozoite, and schizont) labels that had between 60.9 and 78.4% agreement between individual human labels and the majority consensus.

The Angle-PCA projection results provide an ordering along the life cycle continuum, allowing us to compare this sort order to that by human experts. With our ensemble of six models, we could also evaluate the consensus and variation between angle predictions for each example. The consensus between models for relative angle between two examples was greater than 96.6% (and an area under the precision-recall curve score of 0.989; see note S4 for definition), and the median angle variation across all labeled examples was 0.11 radians. The sensitivity of this measurement can be tuned by selecting a threshold for when two parasites are considered equal, resulting in a precision-recall curve (Fig. 2C). When we use the median angle variation of the model as the threshold for examples that are too close to call, we get performance (light blue point) that is representative of the human expert average. These results demonstrate that our semi-supervised model successfully identified and segregated asynchronous parasites and infected RBCs from images that contain >90% uninfected RBCs (i.e., <10% parasitaemia) and classifies parasite development logically along the *P. falciparum* asexual life cycle.

### Quantifying on-cycle drug effects

**Fig. 3:**
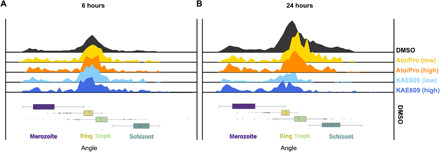
Quantifying on-cycle drug effects. Asynchronous *Plasmodium falciparum* cultures were treated with the ATPase4 inhibitor KAE609 or the combinational MITO treatment of atovaquone and proguanil (Ato/Pro) with samples fixed and imaged 6 (**A**) and 24 (**B**) hours after drug additions. Top panels show histograms indicating the number of parasites across life cycle continuum. Compared to DMSO controls (topmost black histogram), both treatments demonstrated reduced parasite numbers after 24 hours. Shown are four drug/concentration treatment conditions: low-dose Ato/Pro (yellow), high-dose Ato/Pro (orange), low-dose KAE609 (light blue), and high-dose KAE609 (dark blue). Box plots below demonstrate life cycle classifications in the DMSO condition of images from merozoites (purple) to rings (yellow), trophozoites (green), and finishing with schizonts (dark green).

Having demonstrated the semi-supervised model can classify asynchronous life cycle progression consistently with fine granularity, the model was next applied to quantify on-cycle differences (i.e., life cycle stage-specific drug effects) in asynchronous, asexual cultures treated with known antimalarial drugs. Two drug treatments were initially chosen that give rise to aberrant cellular development: the ATP4ase inhibitor KAE609 (also called Cipargamin) (*13*) and the mitochondrial inhibiting combinational therapy of atovaquone and proguanil (*14*) (here referred to as Ato/Pro). KAE609 reportedly induces cell swelling (*15*), while Ato/Pro reduces mitochondrial membrane potential (*16*). Drug treatments were first tested at standard screening concentrations (2 μM) for two incubation periods (6 and 24 hours). Next, drug dilutions were carried out to test the semi-supervised model’s sensitivity to lower concentrations using half-median inhibitory concentrations (IC_50_s) of each compound (table S1). IC_50_ and 2 μM datasets were processed through the semi-supervised model and overlaid onto DMSO control data as a histogram to explore on-cycle drug effects (Fig. 3). KAE609 treatment exhibited a consistent skew toward ring stage parasite development (8 to 12 hours after RBC invasion; Fig. 3) without an increase within this stage of development, while the Ato/Pro treatment led to reduced trophozoite stages (~12 to 30 hours after RBC invasion; Fig. 3). This demonstrates that the fine-grained continuum has the sensitivity to detect whether drugs affect specific stages of the parasite life cycle.

### Identifying off-cycle drug effects on parasite morphology

The improved information granularity was extended to test whether the model could identify drug-based morphological phenotypes (off-cycle) toward determination of MoA. Selecting the penultimate 32-dimensional layer of the semi-supervised model meant that, unlike the Angle-PCA model, outputs were not restricted to discrete on-cycle labels but instead represented both on- and off-cycle changes. This 32-dimensional representation is referred to as the morphology embedding.

Parasites were treated with 1 of 11 different compounds, targeting either PfATP4ase (ATP4) or mitochondria (MITO) and DMSO controls (table S1). The semi-supervised model was used to evaluate three conditions: random, where compound labels were shuffled; Angle-PCA, where the two PCA coordinates are used; and full embedding, where the 32-dimensional embedding was combined with the Angle-PCA. To add statistical support that enables compound level evaluation, a bootstrapping of the analysis was performed, sampling a subpopulation of parasites 100 times.

**Fig. 4:**
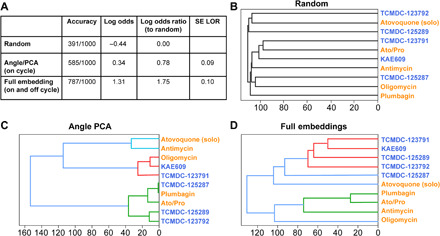
Quantifying off-cycle drug effects. To better define drug effect on *Plasmodium falciparum* cultures, five mitochondrial (orange text) and five PfATP4ase (blue text) compounds were used; after a 24-hour incubation, images were collected and analyzed by the semi-supervised model. To test performance, various conditions were used (**A**). For random, images and drug names were scrambled, leading to the model incorrectly grouping compounds based on known MoA (**B**). Using life cycle stage definition (as with Fig. 3), the model generated improved grouping of compounds (**C**) versus random. Last, by combining the life cycle stage information with the penultimate layer (morphological information, before life cycle stage definition) of the model, it led to correct segregation of drugs based on their known MoA (**D**).

As expected, the randomized labels led to low accuracy (Fig. 4A), serving as a baseline for the log odds (probability). When using the 2D Angle-PCA (on-cycle) information, there was a significant increase over random in the log odds ratio (Fig. 4A). This represents the upper-bound information limit for binary live/dead assays due to their insensitivity to parasite stages. When using the combined full embedding, there was a significant log odds ratio increase over both the random and Angle-PCA conditions (Fig. 4A). To validate that this improvement was not a consequence of having a larger dimensional space compared to the Angle-PCA, an equivalent embedding from the fully supervised model trained only on expert labels (and not on pseudolabels) demonstrated approximately the same accuracy and log odds ratio as Angle-PCA. Thus, our semi-supervised model can create an embedding sensitive to the phenotypic changes under distinct MoA compound treatment.

To better understand drug MoA, we evaluated how the various compounds were grouped together by the three approaches (random, Angle-PCA, and morphology embedding), performing a hierarchical linkage dendrogram (Fig. 4, B to D). The random approach shows that, as expected, the different compounds do not reveal MoA similarities. For the Angle-PCA output, the MITO inhibitors atovaquone and antimycin are grouped similarly, but the rest of the clusters are a mixture of compounds from the two MoA groups. Last, the morphology embedding gave rise to an accurate separation between the two groups of compounds having different MoA. One exception for grouping was atovaquone (when used alone), which was found to poorly cluster with either group (branching at the base of the dendrogram; Fig. 4D). This result is likely explained by the drug dosages used, as atovaquone is known to have a much enhanced potency when used in combination with proguanil (*16*).

The semi-supervised model was able to consistently cluster MITO inhibitors away from ATP4ase compounds in a dimensionality that suggested a common MoA. Our semi-supervised model can therefore successfully define drug efficacy in vitro and simultaneously assign a potential drug MoA from asynchronous (and heterogeneous) *P. falciparum* parasite cultures using an imaging-based screening assay with high-throughput capacity.

## DISCUSSION

Driven by the need to accelerate novel antimalarial drug discovery with defined MoA from phenotypic screens, we applied ML to images of asynchronous *P. falciparum* cultures. This semi-supervised ensemble model could identify effective drugs and cluster them according to MoA, based on life cycle stage (on-cycle) and morphological outliers (off-cycle).

Recent image-based ML approaches have been applied to malaria cultures but have, however, focused on automated diagnosis of gross parasite morphologies from either Giemsa- or Leishman-stained samples (*17*–*19*), rather than phenotypic screening for drug MoA. ML of fluorescence microscopy images have reported malaria identification of patient-derived blood smears (*20*) and the use of nuclear and mitochondrial specific dyes for stage categorization and viability (*21*), although the algorithmic approach did not include deep learning. Previous unsupervised and semi-supervised ML approaches have been applied to identify phenotypic similarities in other biological systems, such as cancer cells (*12*, *22*–*24*), but none have addressed the challenge of capturing the continuum of biology within the heterogeneity of control conditions. We therefore believe our study represents a key milestone in the use of high-resolution imaging data beyond diagnostics to predict the life cycle continuum of a cell type (coping with biological heterogeneity), as well as using this information to indicate drug-induced outliers and successfully group these toward drug MoA.

Through semi-supervised learning, only a small number of human-derived discrete but noisy labels from asynchronous control cultures were required for our DNN method to learn and distribute data as a continuous variable, with images following the correct developmental order. By reducing expert human input, which can lead to image identification bias (see note S2), this approach can control for interexpert disagreement and is more time efficient. This semi-supervised DNN therefore extends the classification parameters beyond human-based outputs, leading to finer information granularity learned from the data automatically through pseudolabels. This improved information, derived from high-resolution microscopy data, permits the inclusion of subtle but important features to distinguish parasite stages and phenotypes that would otherwise be unavailable.

Our single model approach was trained on life cycle stages through embedding vectors, whose distribution allows identification of two readouts, on-cycle (sensitive to treatments that slow the life cycle or kill a specific parasite stage) and off-cycle (sensitive to treatments that cluster away from control distributions). We show that this approach with embeddings was sensitive to stage-specific effects at IC_50_ drug concentrations (Fig. 3), much lower than standard screening assays. Drug-based outliers were grouped in a MoA-dependent manner (Fig. 4), with data from similar compounds grouped closer than data with unrelated mechanisms.

The simplicity of fluorescence imaging means that this method could be applied to different subcellular parasite features, potentially improving discrimination of cultures treated with other compounds. In addition, imaging the sexual (gametocyte) parasite stages with and without compound treatments will build on the increasing need for drugs, which target multiple stages of the parasite life cycle (*25*). Current efforts to find drugs targeting the sexual stages of development are hampered by the challenges of defining MoA from a nonreplicating parasite life cycle stage (*25*). This demonstrates the potential power of a MoA approach, applied from the outset of their discovery, simply based on cell morphology.

In the future, we envisage that on-cycle effects could elucidate the power of combinational treatments (distinguishing treatments targeting different life cycle stages) for a more complete therapy. Using off-cycle, this approach could identify previously unidentified combinational treatments based on MoA. Because of the sample preparation simplicity, this approach is also compatible with using drug-resistant parasite lines.

New drugs against malaria are seen as a key component of innovation required to “bend the curve” toward the disease’s eradication or risk a return to premillennium rates (*3*, *26*). Seen in this light, application of ML-driven screens should enable the rapid, large-scale screening and identification of drugs with concurrent determination of predicted MoA. Since ML-identified drugs will start from the advanced stage of predicted MoA, these should bolster the much-needed development of new chemotherapeutics for the fight against malaria.

## MATERIALS AND METHODS

### Generation of the transgenic 3D7/sfGFP parasite line

To generate parasite line 3D7/sfGFP, 3D7 ring stages were transfected with both plasmids pkiwi003 (p230p-bsfGFP) and pDC2-cam-co.Cas9-U6.2-hDHFR _P230p (50 μg each; fig. S3) following standard procedures (*27*) and selected on 4 nM WR99210 (WR) for 10 days. pDC2-cam-co.Cas9-U6.2-hDHFR _P230p encodes for Cas9 and the guide RNA for the P230p locus. pkiwi003 comprises the repair sequence to integrate into the P230p locus after successful double-strand break induced by the Cas9. pkiwi003 (p230p-bsfGFP) was obtained by inserting two polymerase chain reaction (PCR) fragments both encoding parts of P230p (PF3D7_0208900) consecutively into the pBluescript SK(−) vector with Xho I/Hind III and Not I/Sac I, respectively. sfGFP together with the hsp70 (bip) 5′ untranslated region was PCR-amplified from pkiwi002 and cloned into pkiwi003 with Hind III/Not I. pkiwi002 is based on pBSp230pDiCre (*28*), where the FRB (binding domain of the FKBP12–rapamycin-associated protein) and Cre60 cassette (including promoter and terminator) was removed with Afe I/Spe I, and the following linkers inserted are as follows: L1_F cctttttgcccccagcgctatataactagtACAAAAAAGTATCAAG and L1_R CTTGATACTTTTTTGTactagttatatagcgctgggggcaaaaagg. In a second step, FKBP (the immunophilin FK506-binding protein) and Cre59 were removed with Nhe I/Pst I and replaced by sfGFP, which was PCR-amplified from pCK301 (*29*). pDC2-cam-co.Cas9-U6.2-hDHFR _P230p was obtained by inserting the guide RNA (AGGCTGATGAAGACATCGGG) into pDC2-cam-co.Cas9-U6.2-hDHFR (*30*) with Bbs I. Integration of pkiwi003 into the P230p locus was confirmed by PCR using primers #99 (ACCATCAACATTATCGTCAG), #98 (TCTTCATCAGCCTGGTAAC), and #56 (CATTTACACATAAATGTCACAC; fig. S3).

### *P. falciparum* cell culture

The transgenic 3D7/sfGFP *P. falciparum* asexual parasites were cultured at 37°C (with a gas mixture of 90% N_2_, 5% O_2_, and 5% CO_2_) in human O^+^ erythrocytes under standard conditions (*31*), with RMPI-Hepes medium supplemented with 0.5% AlbuMAX-II. Two independent stocks (culture 1 and culture 2; Fig. 1A) of 3D7/sfGFP parasites were maintained in culture and synchronized separately with 5% d-sorbitol on consecutive days to ensure acquisition of all stages of the asexual cycle on the day of sample preparation. Samples used for imaging derived from cultures harboring an approximate 1:1:1 ratio of rings, trophozoites, and schizonts, with a parasitaemia around 10%.

### Sample preparation and imaging

Asexual cultures were diluted 50:50 in fresh media before 50 nM MitoTracker CMXRos (Thermo Fisher Scientific) was added for 20 min at 37°C. Samples were then fixed in phosphate-buffered saline (PBS) containing 4% formaldehyde and 0.25% glutaraldehyde and placed on a roller at room temperature, protected from light for 20 min. The sample was then washed 3× in PBS before 10 nM DAPI, and WGA (5 μg/ml) conjugated to Alexa Fluor 633 was added for 10 min and protected from light. The sample was then washed 1× in PBS and diluted 1:30 in PBS before pipetting 100 μl into each well of a CellVis (Mountain View, CA) 96-well plate.

Samples were imaged using a Nikon Ti-Eclipse widefield microscope and Hamamatsu electron multiplying charge-coupled device camera, with a 100× Plan Apo 1.4 numerical aperture (NA) oil objective lens (Nikon); the NIS-Elements JOBS software package (Nikon) was used to automate the plate-based imaging. The five channels [brightfield, DNA (DAPI), cytoplasm (sfGFP-labeled), mitochondria (MitoTracker or MITO), and RBC (WGA-633)] were collected serially at Nyquist sampling as a 6-μm z-stack, with fluorescent excitation from the CoolLED light source. To collect enough parasite numbers per treatment, 32 fields of view (sites) were randomly generated and collected within each well, with treatments run in technical triplicate. Data were saved directly onto an external hard drive for short-term storage and processing (see below).

### Image analysis

The 3D images were processed via a custom macro using ImageJ and transformed into 2D maximum intensity projection images. Brightfield channels were also projected using the minimum intensity projection as this was found to improve analysis of the food vacuole and anomalies including double infections. Converting each whole-site image to per-parasite embedding vectors was performed as previously described (*12*), with some modifications: The Otsu threshold was set to the minimum of the calculated threshold or 1.25× of the foreground mean of the image, and centers closer than 100 pixels were pruned. Each channel image was separately fed as a grayscale image into the deep metric network for conversion into a 64-dimension embedding vector. The six embedding vectors (one from each fluorescent channel and both minimum and maximum projections of the brightfield channel) were concatenated to yield a final 384 dimension embedding vector.

### Ground truth

All labels were collected using the annotation tool originally built for collecting diabetic retinopathy labels (*32*). For each set of labels gathered, tiled images were stitched together to create a collage for all parasites to be labeled. These collages contained both stains in grayscale and color overlays to aid identification. Collages and a set of associated questions were uploaded to the annotation tool, and human experts (Imperial College London) provided labels (answers). In cases where multiple experts labeled the same image, a majority vote was used to determine the final label.

Initial labels for training classified parasites into 1 of 11 classes: merozoite, ring, trophozoite, schizont, cluster of merozoites, multiple infection, bad image, bad patch (region of interest) location, parasite debris, unknown parasite inside an RBC, or other. Subsequent labels were collected with parasite debris classified further into the following: small debris remnant, cluster of debris, and death inside a RBC (table S2). For training, the following labels were dropped: bad image, bad patch location, unknown parasite inside an RBC, unspecified parasite debris, and other. For these labels, five parasites were randomly sampled from each well of experiments.

To validate the model performance, an additional 448 parasites were labeled by five experts. The parasites were selected from eight separate experimental plates using only control image data (DMSO only).

Last, paired labels were collected to validate the sort-order results. For these labels, the collage included two parasites, and experts identified which parasite was earlier in the life cycle or whether the parasites were too close to call. Here, data from the 448 parasite validation set were used, limited to cases where all experts agreed that the images were of a parasite inside an RBC. From this set, 24 parasites were selected, and all possible pairings of these 24 parasites were uploaded as questions (24 choose 2 = 276 questions uploaded). In addition, another 19 pairs were selected that were near the trophozoite/schizont boundary to enable angle resolution analysis.

### Data analysis

To prepare the data for analysis, the patch embeddings were first joined with the ground truth labels for patches with labels. Six separate models were trained on embeddings to classify asexual life cycle stages and normal anomalies such as multiple infection, cell death, and cellular debris. Each model was a two-layered (64 and 32 dimensions), fully connected (with ReLu nonlinearities) neural network. To create training data for each of the six models, human-labeled examples were partitioned so that each example within a class is randomly assigned to one of four partitions. Each partition is a split of the data with example images randomly placed into a partition (subject to the constraint that it is balanced for each life cycle category). Each model was then trained on one of the six ways to select a pair from the four partitions. Training was carried out with a batch size of 128 for 1000 steps using the Adam optimizer (*33*) with a learning rate of 2 × 10^−4^. Following the initial training, labels were predicted on all unlabeled data using all six models, and for each class, 400 examples were selected with the highest mean probability (and at least a mean probability of 0.4) and with an SD of the probability less than 0.07 (which encompasses the majority of the predictions with labels). The training procedure was repeated with the original human labels and predicted (pseudo-) labels to generate our final model. The logits are extracted from the trained model, and a subspace representing the normal life cycle stages is projected using 2D by PCA. The life cycle angle is computed as arctan(*y*/*x*), where *x* and γ are the first and second coordinates of the projection, respectively.

For each drug with a certain dose and application duration, the evaluation of its effect is based on the histogram of the classified asexual life cycle stages, and finer binned stages obtained from the estimated life cycle angle. A breakdown of labeled images for drug morphologies is given in table S3.

## Supplementary Material

aba9338_SM.pdf

## SUPPLEMENTARY MATERIALS

Supplementary material for this article is available at http://advances.sciencemag.org/cgi/content/full/6/39/eaba9338/DC1

## Appendix

View/request a protocol for this paper from *Bio-protocol*.
