# Supplementary material for: A machine learning approach to define antimalarial drug action from heterogeneous cell-based screens
Source: Sci Adv. 2020 Sep 25;6(39):eaba9338. doi: 10.1126/sciadv.aba9338 (PMC7518791; doi:10.1126/sciadv.aba9338)
Supplement: aba9338_SM.pdf [file aba9338_SM.pdf]

Science Advances
AAAS

advances.sciencemag.org/cgi/content/full/6/39/eaba9338/DC1

Supplementary Materials for

A machine learning approach to define antimalarial drug action from heterogeneous cell-based screens

George W. Ashdown, Michelle Dimon, Minjie Fan, Fernando Sánchez-Román Terán, Kathrin Witmer, David C. A. Gaboriau, Zan Armstrong, D. Michael Ando*, Jake Baum*

*Corresponding author. Email: mando@google.com (D.M.A.); jake.baum@imperial.ac.uk (J.B.)

Published 25 September 2020, *Sci. Adv.* **6**, eaba9338 (2020)
DOI: 10.1126/sciadv.aba9338

This PDF file includes:

Supplementary Notes 1 to 4
Figs. S1 to S3
Tables S1 to S3

Supplementary Text (Notes 1-4)

Supplementary Note 1: Human expert agreement with majority consensus

To calculate agreement between the six human experts, each labelled image patch was compared to the majority consensus (category with most votes). There was total agreement (5 votes) of lifecycle stage for 23.7 % of parasite images, with 4 or 3 out of 5 votes accounting for 24.4 % and 32.7 % of parasite image classification respectively. Labellers' answers were compared to the majority consensus for 448 images (each expert labelled between 143 and 448 images). Labeller answer versus majority consensus (in descending order of images labelled) was 66.1 %, 78.4 %, 73.1 %, 60.9 %, 71.8 %, 68.8 %.

Supplementary Note 2: Human experts provide noisy initial labels to train supervised models

With typical mammalian cells, almost every normal cell should look approximately the same. Dividing cells look different but are sparse and generally dropped from analysis. *P. falciparum* shows dramatic morphological changes throughout its lifecycle. In order to successfully apply ML, especially when investigating drug induced changes, 'normal' needed to be defined throughout the lifecycle. One solution would be to create synchronized cultures at each stage and use these as the ground truth. In reality, even these synchronized cultures show parasite-to-parasite variability (a 'trophozoite-heavy' culture is still a mix of some late rings, some trophozoites, some early schizonts). Instead, asynchronous cultures were used and collected ground truth labels from human experts. This presents a segregation challenge for the ML when channel intensities range greatly (e.g. DAPI brightness between ring and schizont stages [Figure 1c]). Human labels enable training of a standard supervised random forest model to bin parasites into ring / trophozoite / schizont stages. However, these include increased levels of noise, especially away from canonical images, for example experts disagree about whether a parasite is a late ring or early trophozoite. A random forest trained on these labels also has disagreement with the held-out test dataset. It is unclear if this disagreement is because the human labels are noisy or because the random forest is poorly trained.

Supplementary Note 3: Validating model-derived lifecycle stage ordering

To test the lifecycle continuum defined by the model, 6 human labellers were asked to order pairs of parasite images by labelling the first image as 'earlier' or 'later' in the lifecycle compared to the second image. This demonstrated whether humans and the model correctly ordered parasite stages and to what level of information granularity. Majority consensus was met when 4 of the 6 human labellers agreed on the developmental order of the images. There was also a category for when developmental order was unclear ('too close to call'), this definition was met when labellers' votes were split evenly between 'earlier' or 'later' or when no answer had 3 votes.

Of 295 pairs of images, the majority vote classified 75 as 'too close to call', with 220 classified as 'earlier' or 'later'. Individual human labellers gave a vote of 'earlier' or 'later' between 164 and 219 times, and between 89.5 % and 95.8 % of these labels aligned with the consensus.

Supplementary Note 4: Precision – Recall curve calculations

Precision and recall were calculated against the consensus human paired answers. Precision was calculated as the fraction of before/after images with a definitive answer (i.e. not 'too close to call') that were called correctly. Recall was calculated as the fraction of pairs not called incorrectly that were also given a definitive answer. For the evaluation of the machine learning model, the difference in angle (predicted lifecycle stage) between the two parasite patches was calculated and compared to a 'too close to call' threshold. If the angle difference was less than this threshold, then the ML answer was judged to be 'too close to call'. The curve of precision/recall for the machine learning algorithm was calculated by stepping through this threshold from zero until all pairs were considered too close to call.

Supplementary Figures S1-3

Supplementary Figure S1: Consensus between human labellers and versus machine learning labels

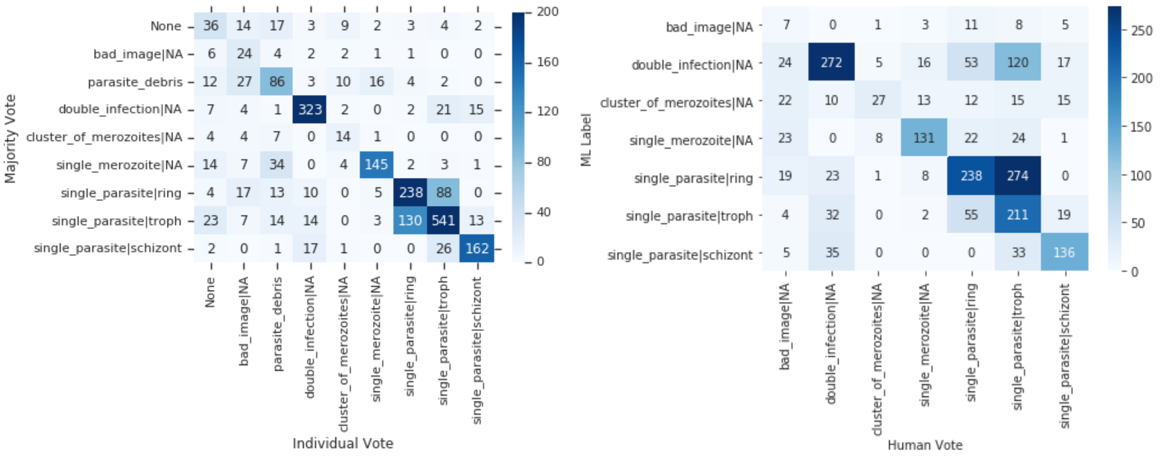

Figure S1. Confusion matrices. A. Confusion matrix demonstrating human labeller consensus versus the majority vote for individual parasite stages. **B.** Confusion matrix demonstrating machine learning labels (pseudolabels) versus human labels consensus.

Supplementary Figure S2: Subset of image patches demonstrating data heterogeneity

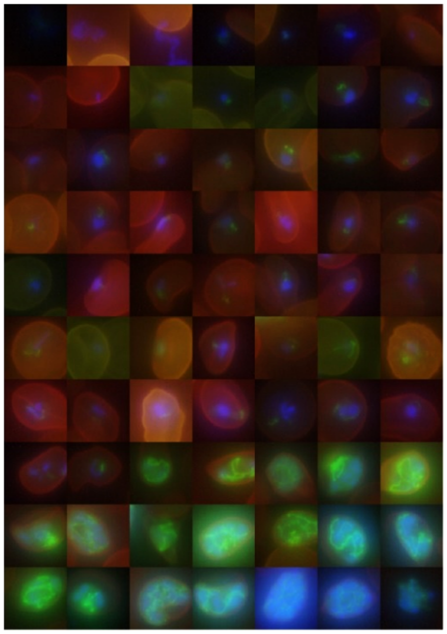

Figure S2. The challenge of dataset heterogeneity. A collage of randomly chosen patches of asexual blood stage parasites, demonstrating the variations in morphology and signal through the lifecycle. The coloured, merged images show red blood cell membrane (red), mitochondria (green) and nucleus (blue).

Supplementary Figure S3: Generation of *P. falciparum* constitutively expressing sfGFP

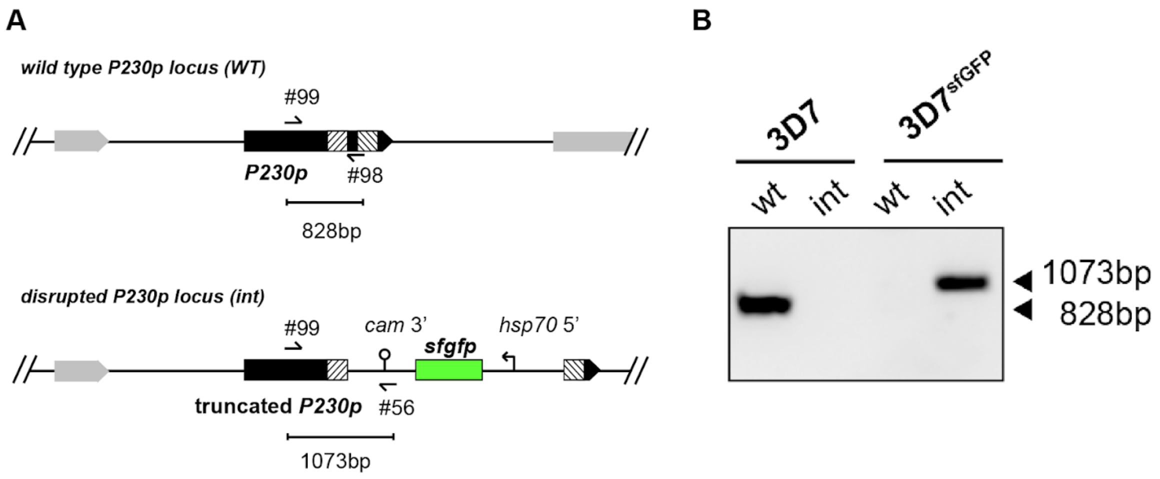

Figure S3. Generating transgenic *P. falciparum* 3D7/sfGFP. A. Schematic map of the *P230p* locus before (WT, top) and after integration of plasmid pkiwi003 (int, bottom). **B.** Integration PCR confirming integration of plasmid pkiwi003 into the P230p locus using primer pairs (#99/98 WT; #99/56 int) from (A).

Supplementary Tables S1-3

Supplementary Table S1. List of drugs used for screening phase.

Drug	Target / Effect	IC50 (nM)	Reference
Atovaquone + Proguanil hydrochloride	Mitochondria Electron Transport chain bc_1 Complex III inhibitor	0.23 + 45 (in combination)	(*16*)
Oligomycin A	Mitochondrial ATP synthase Complex V inhibitor	110	PubChem (*34*)
Atovaquone	Mitochondria Electron Transport chain bc_1 Complex III (Q0 site) inhibitor	0.27	(*35*) and PubChem (*34*)
Antimycin A	Mitochondria Electron Transport chain bc_1 Complex III (Q1 site) inhibitor	614	(*36*)
Plumbagin	Succinate dehydrogenase (SDH) complex II inhibitor	580	(*37*)
KAE609 (cipargamin)	Inhibits ATP4ase, disrupting Na+ regulation in parasites	1	(*15*)
TCMDC-125287	PfATP4ase inhibitor	1700	GSK report on PubChem (*34*)
TCMDC-125289	PfATP4ase inhibitor	50	GSK report on PubChem (*34*)
TCMDC-123791	PfATP4ase inhibitor	970	GSK report on PubChem (*34*)
TCMDC-123792	PfATP4ase inhibitor	200	GSK report on Pubchem (*34*)

Supplementary Table S2. Breakdown for number of lifecycle classifiers per lifecycle stage.

Image label	Count
Single parasite (Merozoite)	226
Single parasite (Ring)	757
Single parasite (Trophozoite)	1075
Single parasite (Schizont)	434
Double infection	1100
Parasite debris	307
Other	146
Bad image	116
Cluster of debris	87
Bad boundary (patch error)	75
Cluster of merozoites	69
Dead parasite (intracellular)	50
Small debris	25
Unlabelled	238,793

Supplementary Table S3. Breakdown for number of cells for drug mechanism of action classification for drug concentrations.

Image label	Drug dose (nM)	Count
Antimycin	2186 / 20000	683 / 495
Atovaquone	0.27 / 2.7	769 / 679
Atovaquone / Proguanil	1000 / 2000	260
DMSO	N/A	1560
KAE609	2 / 20	375 / 415
Oligomycin	111 / 1110	528 / 493
Plumbagin	270 / 2700	843 / 425
TCMDC-123791	970 / 9700	393 / 752
TCMDC-123792	200 / 2000	891 / 386
TCMDC-125287	1700 / 17000	661 / 750
TCMDC-125289	50 / 500 / 2000	742 / 602 / 252
